# An Explanation

**Published:** 1884-05

**Authors:** 


### AN EXPLANATION.

WE ARE frequently in receipt of letters requesting us to write articles upon subjects that are especially within the scope of *medical* publications. The JOURNAL is published for the people at large, and not exclusively for physicians, and in order to write articles that could be comprehended by all our readers, we should be compelled to dispense with technical terms; and to employ plain English in the discussion of certain subjects, would not be in keeping with the claim that has always been made by the JOURNAL—that it never publishes a sentence that could possibly offend the most sensitive reader.

While we cannot consistently accede to these requests, we deeply sympathize with those who suffer, mentally or bodily, even though the responsibility of these ills may rest with themselves.

The popularity of The JOURNAL OF HEALTH has caused its Editor to be made the confidant of scores of persons who, with reason or without it, believe themselves to be sorely afflicted.

It is, perhaps, premature to announce the publication of a pamphlet that we have long contemplated writing for their benefit; but in the meantime we earnestly advise every one who has a well-grounded suspicion of any disease, of whatever nature, to personally consult a competent physician, without delay. As a rule, all fees sent to persons who advertise that they cure diseases, might better be burned up, since the injury they do the patient is usually greater than the loss of his money

